# Accuracy of Triage Systems in Disasters and Mass Casualty Incidents; a Systematic Review

**DOI:** 10.22037/aaem.v10i1.1526

**Published:** 2022-04-30

**Authors:** Jafar Bazyar, Mehrdad Farrokhi, Amir Salari, Hamid Safarpour, Hamid Reza Khankeh

**Affiliations:** 1 Health in Emergency and Disaster Research Center, University of Social Welfare and Rehabilitation Sciences, Tehran, Iran.; 2 Department of Nursing, School of Nursing and Midwifery, Ilam University of Medical Sciences, Ilam, Iran.; 3 Pre-hospital Medical Emergency organization, Ilam university of Medical Sciences, Ilam, Iran.; 4 Department of Health in Emergencies and Disasters, School of Public Health, Tehran University of Medical Sciences, Tehran, Iran.; 5 Non-Communicable Diseases Research Center, Ilam University of Medical Sciences, Ilam, Iran.

**Keywords:** Disasters, Data Accuracy, Triage, Mass Casualty Incidents, Systematic review

## Abstract

**Introduction:**

To prioritize patients to provide them with proper services and also manage the scarce resources in emergencies, the use of triage systems seems to be essential. The aim of this study was to evaluate the accuracy of the existing triage systems in disasters and mass casualty incidents.

**Methods:**

The present study is a systematic review of the accuracy of all triage systems worldwide. The results of this study were based on the articles published in English language journals. In this research, all papers published from the beginning of 2000 to the end of 2021 were sought through different databases. Finally, a total of 13 articles was ultimately selected from 89 articles.

**Results:**

13 studies on the accuracy of triage systems were reviewed. The START, mSTART, SALT, Smart, Care Flight, ASAV, MPTT, Sieve and ESI triage systems, had an accuracy, sensitivity, and specificity of less than 90%. Only the Smart triage system had an overall accuracy of more than 90%.

**Conclusion:**

According to the findings of the current systematic review, the performance of the existing triage systems in terms of accuracy of prioritizing the injured people and other performance indexes is not desirable. Therefore, to improve the performance and increase the precision of triage systems, the world nations are recommended to change or revise the indexes used in triage models and also identify other influential factors affecting the accuracy of triage systems.

## 1. Introduction:

Triage, with a French origin (Trier), means *prioritizing* and is used to classify patients and people affected by emergencies and disasters. This classification leads to better management of services and optimal use of the available resources for injured people and patients (1). A triage system is considered optimal, when it can identify patients and injured individuals who need immediate care and provide access to rapid diagnostic and therapeutic measures (2, 3). If the triage system does not function properly and categorizes patients appropriately, it will cause waste of resources, delay in the provision of services to patients according to their needs, dissatisfaction and adverse outcomes in patients, and in some cases, it may endanger the patient’s life (4). Thus, the use of an efficient triage system with good reliability and validity seems to be essential when providing services to patients and injured individuals, which sometimes determines the survival of these people (5-7). The inhabitants of the world are annually faced with many natural and man-made hazards and emergencies, which have caused a lot of physical damage to these people (8). The use of the triage system for the people who have been injured because of emergencies and disasters that harm a large number of people is considered a necessary measure, through which appropriate health care and survival of the injured can be guaranteed (9-11).

Nowadays, to prioritize patients, different triage systems are applied based on the age group, the cause of damage, the geographical area, and other characteristics of the affected people (1). The most common of which are Simple Triage and Rapid Treatment (START), Modified Simple Triage Algorithm and Rapid Treatment (mSTART), Sort, Assess, Lifesaving interventions, Treatment/Transport (SALT), SMART, Care Flight, Amberg-Schwandorf Algorithm for Primary Triage (ASAV), Modified Physiological Triage Tool (MPTT), Sieve**, **and Emergency Severity Index (ESI) triage systems. These triage systems are increasingly being used to prioritize injured people in emergencies and disasters, as well as in health and medical systems; however, there is no single comprehensive system that is universally agreed upon. Each country or region of the world uses a different system for triage based on its own needs (12-15). In general, there are many controversies in the world concerning the accuracy of the triage systems. Therefore, to determine the proper functioning of these triage systems, their accuracy is evaluated using several divergent indicators encompassing sensitivity, specificity, positive and negative predictive value, overall accuracy, over-triage, and under-triage (16, 17). Sensitivity and specificity indicate the correct classification of the affected people and the predictive value of the triage indicates the power of the triage method in correct classification of these individuals. Over-triage categorizes injured individuals or patients in higher classes and under-triage in lower classes compared to their actual level of severity. In over-triage, the person is provided with a service that he/she does not need, resulting in waste of time and resources, on the contrary, in under-triage, the person is provided with services not meeting his actual needs, which can endanger the life of the individual (17, 18).

Various studies have been conducted to estimate the accuracy of different types of triage systems worldwide, but, to the best of our knowledge, there is no comprehensive study on the comparison of different types of triage methods. In other words, in the studies done, only two or three methods have been compared. Consequently, the present study aims to assess and compare the accuracy of the indicators of the all triage systems through a systematic review.

## 2. Methods:

***2.1. Study protocol and Search strategy***

The present study is a systematic review of the accuracy of the all triage systems used worldwide. The results of this study were based on the articles published in English language journals. In this research, all papers published from the beginning of 2000 to the end of 2019 were sought through the Medlib, Scopus, Web of Science, PubMed, Cochrane Library, Science Direct, and Google scholar databases. All articles with medical subject headings (MeSH) and keywords including triage systems, sensitivity, specificity, predictive values, over-triage, under-triage, disaster, and mass casualty incidents (MCIs) were searched; the keywords were used in isolation or in combination, using and/or: Triage systems OR disaster OR mass casualty incidents AND accuracy OR sensitivity OR specificity OR predictive values OR over triage OR under triage.

***2.2. Screening and Data extraction***

Accordingly, all articles on triage systems were first collected and, upon completion of the search, a list of abstracts was prepared. After concealing the profile of the articles, such as the name of the author, the name of the magazine, etc., the full text of the articles was given to two qualified researchers to review the articles. Each article was independently reviewed by two people. If the articles were rejected by the two reviewers, the reason was also mentioned by them and in case of disagreement between them, the article was judged by a third reviewer. Data extraction was performed using a pre-prepared checklist that included the study location, study time, triage system, community under study, sensitivity, specificity, predictive value, over-triage, and under-triage. 

***2.3. Quality assessment***

The quality of articles was assessed using the Newcastle Ottawa Scale (NOS) checklist. This checklist includes 8 items, each ranging from 0 to 1. The scores between 0 to 5, 6-7, and 8 represent low, medium, and high quality, respectively. The lowest acceptable score for entering the study was 5 (19). 

***2.4. Inclusion and exclusion criteria***

Articles meeting all the following criteria were included in the study: addressing Triage systems; reporting one or all of following indexes including validity and reliability (sensitivity, specificity, positive and negative predictive values, over-triage, and under-triage); having high quality according to the NOS checklist, and being published in English. Interventional studies, conference proceedings, qualitative studies, and case reports, as well as studies not written in English were excluded.

***2.5. Study selection***

**Diagram 1:**
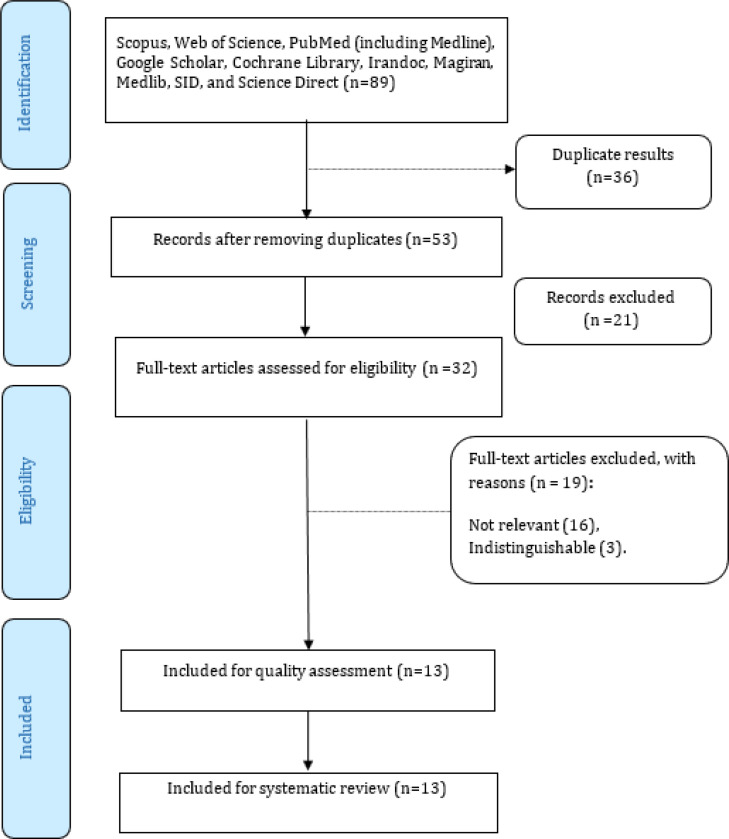
The PRISMA flow diagram of the present study

There were 89 papers on triage systems, out of which, 36 and 21 articles were excluded due to repetitiveness and irrelevance to the study, respectively. After reviewing the abstract of the articles, 19 additional papers lacking the required information were excluded from the study. Finally, 13 articles, which met the inclusion criteria, were considered (Diagram 1).

**Triage systems in results**

Different studies have been done to evaluate the validity and reliability of the triage systems, with the aim to measure the accuracy of these systems in the evaluation of the injured people. Typically, the basics of performing triage systems are similar. So, a triage system should be used, which could recognize and prioritize the injured people, take less time, and manage resources based on the patients' needs. The accuracy of the triage systems, which is evaluated using sensitivity, specificity, positive and negative predictive value, as well as the amount of over- and under-triage, both of which affect the efficient use of limited resources, can show the performance of the triage system in accurate prioritization of the patients and optimal use of the available resources. Accordingly, the present study aims to probe the accuracy of nine world-class triage systems, including START, mSTART, SALT, Smart, Care Flight, ASAV, MPTT, Sieve and ESI. The characteristics of the reviewed articles are presented in Table 1. 

**Triage Systems in the Prehospital Setting**

***The START Triage system***: This is one of the most common triage systems in large events such as natural disasters, used for people over 8 years of age. The duration of this triage is about 30 to 60 seconds. In this method, people are classified with green (minor injury), yellow (delayed), red (immediate) and black (died) tags, and the indicators under investigation in this system are the ability to walk, breathing and its rate, capillary refill time, and the ability to follow commands (1, 17, 20).

***The mSTART triage system***: This triage system is a modulated type of the START triage system whose performance and indices are similar to START triage system. The only difference is that, in the mSTART method, the capillary refill time index is replaced by no palpable pulse index (21).

***The SALT triage system: *** Similar to the START method, in this method, individuals are categorized using green, yellow, red, and black tags, but people are prioritized in a different way. In the SALT method, people are firstly classified into three groups based on walking ability. Then, based on the prioritization performed in the first stage, and with the aim of controlling life-threatening factors, the second step is to evaluate and take actions such as severe bleeding control, airway opening, chest compression, and antidote injection. Finally, following the treatment and response of the injured people to the treatment, they will be prioritized using one of the tags mentioned above, and then treated and transmitted to medical centers (1, 5, 22-24).

***The Smart Triage System*** *:* This triage system, like other systems, has 4 tags and determines priorities based on walking, breathing, capillary refill time, and the ability to follow the commands (25).

***The Care Flight triage system*** *:* In the Care Flight triage method, people are assessed on the basis of walking ability, respiration, radial pulse, and the ability to follow the commands using 4 green (delayed), yellow (emergency), red (immediate) and black (unsalvageable) tags, with the difference that the criterion concerning “following commands” is checked before other criteria. This triage system is used to quickly target a large number of casualties and takes about 15 seconds to complete the triage (1).

***The ASAV Triage System***: This triage system is a new triage method. Individuals, as in other triage methods, are classified via 4 tags including green (minor injury), yellow (delayed), red (immediate) and black (died). In this system, people are classified according to the severity of their bleeding, respiration, radial pulse, and the ability to follow commands (1, 26).

***The Sieve Triage System*** *:* This method is used for adults. In this method, people are prioritized using four colors: green, yellow, red, and black. The indicators investigated in this method are walking, respiration, and pulse rate (5).

***The MPTT Triage System*** *:* In this method, people are prioritized using 4 colors including green, yellow, red, and black. Indicators examined in this method include the ability to walk, breath, pulse, and Glasgow coma scale (GCS), indicating the level of consciousness of the person.

**Triage Systems in Hospital Setting**

***The 5-level triage system (ESI) (Emergency severity index)*** *:* This is one of the most widely used triage systems, which is currently accepted by many countries around the world (26). Although the ESI is the most commonly used method for routine triage, this method is based both on acuity and resource needs, and could also theoretically be used in MCI situations to prioritize patients for placement in the hospital (27). In this system, patients are classified into five levels based on the severity of illness or injury and the need for facilities. At levels 1 and 2, injured patients are prioritized based on the level of severity and at levels 3, 4 and 5, based on the need for emergency facilities. In this method, injured individuals have suffered the most harm at level 1, and the least injury at level 5 (20).

## 3. Results

13 articles published between 2000 and 2021 in the field of triage systems around the world were included in this study, in which the accuracy of triage systems was investigated. In this study, the accuracy of triage systems has been assessed in terms of sensitivity, specificity, positive and negative predictive values, over-triage, under-triage, and overall accuracy. 

***3. 1. The accuracy of the START Triage System***:

 In the study conducted by Mary Colleen Bhalla et al. in the United States (2015) on 100 patients aged 17 to 92, who were traumatized by incidents and emergencies and evaluated using the START triage system, the accuracy of the START triage system was measured. In this study, after evaluating the patients, the sensitivity and specificity for accuracy of triage were 55% and 85%, and the level of over-triage and under-triage was 12% and 33%, respectively. Furthermore, based on the prioritization and the tags assigned to the patients, sensitivity, specificity, and positive and negative predictive values were 80%, 55%, 72.4%, and 79.2% for the green color (minor injury), respectively. These indicators were 0%, 76.8%, 0%, and 93.6%, for the yellow color (delayed). They were also 13.8%, 93%, 44.4%, and 72.5%, and 50%, 100 %, 100%, and 96.9% for the red (immediate) and black colors (died), respectively (16).

 CA Kahn et al. (2003) performed a study in California on 148 people injured in rail accidents. In that study, they used the START Triage system to prioritize the injured patients. They have reported that the degree of sensitivity and specificity of triage has been equally 90%, and the prioritizing accuracy of the injured patients has been 44.6%. In addition, 79 (53%) and 3 (2%) cases were subjected to over-triage and under-triage, respectively. In this study, based on the priority assigned to the injured people, sensitivity, specificity, positive and negative predictive value were 45.8%, 89.3%, 94.8%, and 27.8%for the green priority 39.1% %, 11.9%, 13.2%, and 36.4% for the yellow priority and 100%, 77.3%, 9.1%, and 100% for the red priority (17).

 In another study conducted by Alan Garner et al. in Australia on 1144 injured people, who were injured due to various causes, including road-traffic accident, industrial, sports, burn, etc. incidents, the START triage system was used to prioritize patients and its accuracy was evaluated. Accordingly, the sensitivity of this method was 85% and the specificity level was 86% (21).

In a study conducted by Wallis et al. (2006), sensitivity and specificity were estimated to be 39.2% and 78.7%, respectively. In a study conducted by McKee et al. (2019) on 125 subjects, correct triage rate was 36%, over-triage 7.2% and under-triage 56.8% (28).

In the study conducted by France et al., who aimed to assess the diagnostic accuracy of the START algorithm for disaster triage, the results showed that proportion of patients correctly triaged using START ranged from 0.27 to 0.99 with an overall triage accuracy of 0.73 (95% CI, 0.67 to 0.78). Proportion of over-triage was 0.14 (95% CI, 0.11 to 0.17) while the proportion of under-triage was 0.10 (95% CI, 0.072 to 0.14)(29).

***3. 2. The accuracy of the mSTART triage system***

In a study conducted by Garner et al. in Australia (2001) on 1144 people, who were injured due to traffic, industrial, sports, burn, etc. incidents, which addressed the accuracy of the mSTART triage system, the sensitivity and specificity of the system were estimated to be 84% and 91%, respectively(21). 

In another study, Philipp Wolf et al. in Germany (2014) used the mSTART triage system to prioritize 780 injured patients and assessed the validity and reliability of this method. The sensitivity and specificity of this method were 88.2% and 93.9%, respectively. Moreover, the accuracy of the prioritization of the patients was 84.8% and over-triage and under-triage levels were 3.8% and 6.8%, respectively. It is worth mentioning that the mean duration of the triage was 41 sec in each patient (26).

***3. 3. The accuracy of the SALT triage system***

Mary Colleen Bhalla et al., in a study conducted in the United States (2015), on 100 patients, who were traumatized by accidents and emergencies and evaluated using SALT triage system, estimated the sensitivity and specificity of the SALT triage system to be 65% and 88.3%, respectively. In this study, over-triage and under-triage were 5% and 30%, respectively. Moreover, based on the prioritization of the patients, sensitivity, specificity, and positive and negative predictive values were 91.7%, 47.5%, 72.4%, and 79.2% for the green color (minor injuries), 20%, 93%, 54.5%, and 74.2% for the yellow color (delayed), 7.20%, 93%, 54.5%, and 74.2% for the red color (immediate), and 50%, 100%, 100%, and 9.96% for the black color (died), respectively(16). 

In another study performed by Cone DC in the United States (2011) on 547 injured people, who were injured in highway traffic accidents, the SALT triage system was used to prioritize the injured patients. In this study, the overall accuracy of triage of the injured people was equal to 70%. Over-triage and under-triage were also 6.8% and 23.2%, respectively (25).

 In a scenario study in Alabama, Jones et al. (2014) investigated the accuracy of SALT triage system. The accuracy, over-triage, and under-triage were estimated to be 66%, 22%, and 10%, respectively. In a study conducted by McKee et al. (2019) on 125 subjects, correct triage rate was 52%, over-triage 21.6%, and under-triage was 26.4% (28).

***3. 4. The accuracy of the Smart Triage System***

 In a study done by Cone DC et al. (2011) in the United States, which investigated the accuracy of the Smart Triage system on 544 highway accident victims, the overall accuracy of the prioritization of the system was 93%. Over-triage and under-triage rates were 1.8% and 5.1%, respectively (25).

***3. 5. The accuracy of the Care Flight triage system:***

Garner et al., in a study conducted in Australia (2001), used the Care Flight Triage system to prioritize 1144 individuals injured by traffic, industrial, sports, burn, etc. incidents. Subsequently, the validity and reliability of this triage system were evaluated. Accordingly, the sensitivity and specificity of this method were 82% and 96%, respectively (21). 

In their study, Wallis and Carley, estimated the accuracy and specificity of Care Flight Triage system %98.9 and %39.2, respectively. In a study conducted by McKee et al (2019) on 125 subjects, correct triage rate was 36%, over-triage 5.6%, and under-triage was 57.6% (28).

***3. 6. The accuracy of the ASAV Triage System***

 In a study performed by Philipp Wolf et al. in Germany (2014), which prioritized 780 injured victims using the ASAV triage system, and assessed the validity and reliability of the system, the sensitivity and specificity of the system were 87.4% and 91%, respectively. Furthermore, the overall accuracy of the prioritization of casualties was 83.9%. The over-triage and under-triage rates were 4.6%, and 9.7%, respectively. In this method, the average time required for the triage of each injured person was 35.4 seconds (26).

***3. 7. The accuracy of the MPTT Triage System***

 In a study done by James Vassallo et al., in England (2017), on 5654 injured people over 18 years of age, the MPTT triage system was used to prioritize the injured participants and the accuracy of the triage system was evaluated. The sensitivity and specificity levels of the MPTT triage system were 69.9% and 65.3%, respectively (30).

***3. 8. The accuracy of the Sieve Triage System***

In a study conducted by Alan Garner et al. in Australia (2001), which investigated the accuracy of the Sieve triage system, the sensitivity and specificity of this triage system were estimated to be 45% and 88%, respectively. In this study, the use of capillary refill time or heart rate index for determining the index of pulse rate and prioritization of the casualties was also investigated. Results revealed that there was no significant difference between sensitivity and specificity levels, when the two methods were examined. Particularly, when capillary refill time index was used, the sensitivity and specificity were 45% and 89%, respectively. They were 45% and 88%, respectively, when the heart rate index was taken into consideration (21). In a study conducted by McKee et al. (2019) on 125 subjects, correct triage rate was 36.8%, over-triage 6.4%, and under-triage 57.6% (28).

***3. 9. The accuracy of the 5-level triage system, or ESI***

The validity and reliability of the ESI triage system was investigated in a study conducted by Kariman et al. in Iran (2011) on 1050 patients. In this study, the sensitivity and specificity of the prioritization of the patients were 100% and 99.8% at level 1, 53.2% and 97.5% at level 2, 90.7% and 93.7% at level 3, 67.1% and 98.3% at level 4, and 98% and 94% at level 5, respectively (20). In another study done by Buschhorn BH et al. in the United States (2013) on 150 patients, the validity and reliability of the ESI triage system were evaluated. In this study, sensitivity, specificity, and the overall accuracy of prioritizing the patients were 0%, 97.3%, and 94.7% at level one, 57.1%, 84.9%, and 69.3% at level two, 67.9%, 68.1%, and 68% at level 3, and 33.3%, 93.1% and 90.1% at level 4, respectively. At level 5, only specificity value was estimated, which was equal to 96% (31). In a study conducted by Platts-Mills TF et al. in Carolina (2010), 782 patients were evaluated using ESI triage system. In this study, the validity and reliability of the ESI triage system were investigated in the first level, which showed a sensitivity and specificity of 42.3% and 99.2%, respectively. In this study, the overall accuracy of patient prioritization was 40% in all patients, and 85% at the first level (32). The accuracies of the studied triage systems, based on the results of the systematic review, have been shown in table 2.

## 4. Discussion:

The accuracy of the triage system indicates that the injured people have been classified properly, based on the severity of the injury, to receive medical services. So, misclassification of injured persons will lead to ineffective usage of medical resources and may cause avoidable death. The following criteria can be applied to determine the accuracy of triage systems such as sensitivity, specificity, positive/negative predictive value, and over-triage/under-triage. The sensitivity and specificity indicators show the correct prioritization of the injured and the predictive value indicates the power of the triage method in correct classification of these individuals. Over-triage and under-triage would put the injured people in a wrong group compared to their real level of injury. This depends on the sensitivity and specificity levels of the triage system. Over-triage will lead to provision of services more than what is needed for the patients, which in turn can lead to a waste of time and resources. On the contrary, under-triage, can lead to provision of less health services than needed, which can endanger the life of the person. Although according to the previous studies, 50% over-triage and 5% under-triage are acceptable, needless to say, it is better to reduce over-triage and especially under-triage as much as possible (16, 18, 25, 26, 30). The closer the accuracy of a triage system is to 100%, it means that the patient is properly placed in the desired category and no patient is over-triaged or under-triaged, which is the ideal. When over-triage and under-triage are zero, the triage accuracy is 100% and no patient is missed or no resources are wasted. This is an ideal case, but triage systems are affected by various factors, such as the person who performs triage or the system and accuracy of triage tools, over-triage should be increased so that the disease does not get worse and under-triage goes to zero. 

In the present study, the range of accuracy was wide in all triage tools. The accuracy and effectiveness of MCI triage systems’ analysis is limited, because there are no gold standard definitions for each triage category. Unless there is agreement on which patients should be categorized in each triage category, it will be impossible to calculate the sensitivity and specificity or to compare the accuracy between the triage systems (33).

In mass casualty incidents, the great number of injured patients, the limited resources (for example few professional health care providers and medical equipment), urgent need for medical services, and delay in provision of health services can endanger the lives of the injured patients (34). It should be mentioned that many factors can influence over-triage and under-triage (35). One of the prominent factors affecting the extent of over-triage and under-triage is the level of triage experts’ related knowledge and experience. In fact, if the triage performers do not possess the necessary training background and experience, the injured people will fall into wrong categories and hence, over-triage and under-triage rates will increase. The important issue is the situation in which the triage system is evaluated, either a scenario or real events, which are different and the person doing the triage is differently affected by triage error (35). The person doing the triage in a scenario situation has less environmental stress and is less affected by a triage error (36, 37). In addition, the person already has the necessary training and preparation for triage and his/her performance has improved, which results in improved triage accuracy (37, 38). 

Other factors that affect the level of over-triage and under-triage include the type and the location of the incident, and the type of injury. The mechanism of the injury, affects the severity of injuries. As a result, if an incident, such as suicide, causes serious injuries to people, injured people are usually severely damaged and they are placed in the immediate or dead category. As for the type of injury, if the incident causes internal injury such as internal bleeding, or liver or spleen rupture, it may be impossible or difficult to detect these injuries using anatomical or physiological findings. So the injured person is placed in the wrong category and it will eventually result in an increase in over-triage or under-triage. The place of the event also affects the amount of over-triage and under-triage. If, for example, the event takes place in urban places, due to the availability of advanced medical equipment and services for detecting injuries, over-triage and under-triage rates decrease, whereas in rural and distant places, due to the lack of access to such equipment, over-triage and under-triage rates increase. In general, the time and amount of access to specialized medical equipment, especially in re-triage, affect over-triage and under-triage. When an incident occurs in the urban area, since the patient enters the specialized emergency system quickly and is treated quickly, he/she is less affected by the change in triage level. However, when the patient is far from a specialized center, such as in rural or road areas, the patient's triage level changes because he/she may not receive appropriate treatment. Finally, it can be said that a good triage system should have a high accuracy with the lowest level of over-triage and under-triage. One possible solution to increase the accuracy of triage systems is to increase the number of re-triage steps. Increasing the number of re-triage steps can reduce over-triage and subsequently increase the triage accuracy, but conversely, it can increase under-triage and increase the duration of triage (17, 39, 40). 

The triage systems in different conditions do not use the same unit standards, which leads to achievement of different accuracies. To test the accuracy of triage systems around the world, there are two gold standards as references, and all triage systems are currently compared to them (35). The most important factor in classifying casualties is the severity of the injury and the possible consequences of death and recovery.

In general, for determining the accuracy of triage systems, indicators such as sensitivity, specificity, positive and negative predictive values, overall accuracy, over-triage, and under-triage are used. Once the triage systems are functioning properly, and the rates of over-triage and under-triage are low; however, some references have mentioned that even up to 50% over-triage is acceptable and under-triage should be less than 5% (41). Sensitivity indicates the precise classification of the patient and specificity indicates the classification of healthy people in the correct category, both of which are closely related to the level of over-triage and under-triage. Finally, a system is considered a desirable triage system, when it has an acceptable level of accuracy and can prioritize the patients in the correct order. As a result, the triage systems used in different parts of the world should be assessed for validity and reliability, and especially for accuracy (33). Then, in case of deficiencies in the performance of these systems, their indicators could be corrected or changed, so that the accuracy level of these systems in patient prioritization would reach the optimal level. By doing so, the injured people will receive the required services without any problem, and even in some cases, their lives will not be compromised.

Triage tools are composed of different indicators. How accurate an indicator is and how professionally it assesses the condition of the patient or the injured affects triage error. Sometimes a triage system is designed for emergencies and disaster situations. It is natural that we will move to a triage system that is simple, convenient, and not complicated. This simplicity affects triage error. In addition, since time is valuable, we must accept the percentage of triage error so that we can prioritize the casualty more easily and quickly, which in turn leads to triage error. Conversely, when we are in normal and routine conditions, we need a more accurate triage system because patients are triaged one by one and there is no problem in terms of resources, equipment and manpower, and it is natural that the rate of under-triage and over-triage in these conditions is lower. Therefore, one of the factors that causes differences in triage tools is what indicators that tool consists of and whether it is designed for disaster conditions or routine conditions. Secondly, the factor that greatly affects the differences between triage systems is that, unfortunately, many of the tests that have been performed have not used the reference gold standard (33).

The error related to the triage tool is due to the indicators in the tool. For example, in the START triage, the breathing and pulse index have no range, but in the Sieve and Sort triage, there is a specific range. Also, in the START triage, the walking wound index has no classification and only evaluates the ability to walk and not walk, but in the Sieve tool, this indicator has three ranges, which can affect the accuracy of the triage tool (35). Another issue is the allocation of the injured to different color categories, especially in testing the tool in scenario conditions, which affects the accuracy of the tool. For example, the START triage tool may be better at identifying the injured in the green group than in the red and yellow groups. The accuracy of this tool will automatically increase. Unfortunately, there is no standard for allocating casualties to the categories (33, 35).

**Table1:** General characteristics of the studied articles that were eligible for the systematic review

**Author**	**System**	**Abstract finding**	**Study quality** **(NOS)**
Mary Colleen Bhalla(16); USA; 2015	SALT, START	The mechanism of injury was 41% motor vehicle collision, 32% fall, and 16% penetrating trauma. Hospital outcome was 60% minor/green, 5% delayed/yellow, 29% immediate/red, and 6% dead/black. The SALT method resulted in 5 over-triaged patients, 30 under-triaged, and 65 met triage level. The START method resulted in 12 over-triage, 33 under-triaged, and 55 at triage level. Within triage levels, sensitivity ranged from 0% to 92%, specificity from 55% to 100%, positive predictive values from 10% to 100%, and negative predictive value from 65% to 97%.	High
Christopher A. Kahn(17); California; 2009	START	Field triage designations comprised 22 red (immediate), 68 yellow (delayed), and 58 green (minor) patients. Outcome-based designations found 2 red, 26 yellow, and 120 green patients. Seventy-nine patients were over-triaged, 3 were under-triaged, and 66 patients’ outcomes matched their triage level. No triage level met both the 90% sensitivity and 90% specificity requirement set forth in the hypothesis; yet, red was 100% sensitive and green was 89.3% specific.	High
David C. Cone(25); Sweden; 2011	SALT, Smart	The students had a mean triage accuracy of 70.0% with SALT versus 93.0% with Smart (P =0.0001). Mean over-triage was 6.8% with SALT versus 1.8% with Smart (P = 0.0015), and mean under-triage was 23.2% with SALT versus 5.1% with Smart (P = 0.0001).	High
Alan Garner(21); Australia; 2001	CareFlight, START, mSTART, Sieve	The differences between CareFlight Triage, Simple Triage and Rapid Treatment, and modified Simple Triage and Rapid Treatment were not dramatic, with sensitivities of 82%, 85%, and 84%, respectively, and specificities of 96%, 86%, and 91%, respectively.	High
Philipp Wolf(26); Germany; 2014	ASAV	For red patients, sensitivity of ASAV was 87%, specificity 91%, over-triage 6%, and under-triage 10%. There were no significant differences between ASAV and mSTART. ASAV triage required a mean of 35.4 sec per patient.	High
JamesVassallo(30); United kingdom; 2017	MPTT	The MPTT had a sensitivity of 69.9% and specificity of 65.3%, and showed an absolute increase in sensitivity over existing tools ranging from 19.0% (Modified Military Sieve) to 45.1% (Triage Sieve).	High
Kariman H(20); Iran; 2013	ESI	The sensitivity of triage for steps I, II, III, IV and V was 100%, 53.2%, 90.7%, 67.1%, and 98%, respectively. The specificity of triage for steps I, II, III, IV and V was 99.8%, 97.5%, 93.7%, 98.3%, and 94%, respectively.	Moderate
Buschhorn BH(31); USA; 2013	ESI	For ESI level 1, EMS providers were 0% sensitive and 97.3% specific. They were 94.7% accurate in their assignments of patients to, or not to, ESI level 1. For ESI level 2, the EMS providers were 57.1% sensitive and 84.9% specific. Their overall accuracy in assigning patients to, or not to, ESI level 2 was 69.3%. In ESI level 3, sensitivity was 67.9% and specificity was 68.1%. The accuracy of the prehospital providers in assigning patients to, or not to, ESI level 3 was 68.0%. In ESI level 4, prehospital provider sensitivity was 33.3%, specificity was 93.1%, and accuracy was 90.1%.	Moderate
Platts Mills TF(32); Carolina; 2010	ESI	The sensitivity of ESI in identifying patients in need of receiving an immediate intervention was 42.3% (95% confidence interval [CI] = 23.3% to 61.3%); the specificity was 99.2% (95% CI = 98.0% to 99.7%).	High
Wallis LA(42); South African; 2006	Careflight, JumpSTART, START	Overall, the Careflight score had the best performance in terms of sensitivity and specificity. The performance of the PTT was very similar. In contrast, the JumpSTART and START scores had very low sensitivities, which meant that they failed to identify patients with serious injuries, and would have missed the majority of seriously injured casualties in the models of major incidents.	High
Jones N(43); Alabama; 2015	SALT, JumpSTART	Forty-three paramedics were enrolled. Seventeen were assigned to the SALT group with an overall triage accuracy of 66% ±15%, a mean over-triage rate of 22 ± 16%, and an under-triage rate of 10 ± 9%. Twenty-six participants were assigned to the JumpSTART group with an overall accuracy of 66 ± 12%, a mean over-triage rate of 23 ±16%, and an under-triage rate of 11.2 ± 11%.	High
McKee(28); USA; 2019	START, SALT, Sieve, Careflight	We found that SALT triage most often correctly triaged adult emergency department patients compared to a previously published criterion standard.	Moderate
France J.(29); Global(Systematic review); 2021	START	Proportion of victims correctly triaged using START ranged from 0.27 to 0.99 with an overall triage accuracy of 0.73 (95% CI, 0.67 to 0.78). Proportion of over-triage was 0.14 (95% CI, 0.11 to 0.17), while the proportion of under-triage was 0.10 (95% CI, 0.072 to 0.14). There was significant heterogeneity among the studies for all outcomes (P < .0001).	High

START: Simple Triage and Rapid Treatment; mSTART: Modified Simple Triage Algorithm and Rapid Treatment; SALT: Sort, Assess, Lifesaving interventions, Treatment/Transport; ASAV: Amberg-Schwandorf Algorithm for Primary Triage; MPTT: Modified Physiological Triage Tool; ESI: Emergency Severity Index.

**Table 2:** The Accuracy of triage systems based on the results of the systematic review

**Systems**	Articles*	**Accuracy# **	**Sensitivity **	**Specificity **	**Over-triage **	**Under-triage **
START	6	36 -73	39.2 – 90	78.7 - 90	12 - 53	2 - 33
mSTART	2	84.8	84-88.2	91-93.9	3.8	6.8
SALT	4	66 - 70	65	88.3	5 - 22	10 - 30
Smart	1	93	-	-	1.8	5.1
Care Flight	3	36	39.2-96	96 - 98.8	5.6	57.6
ASAV	1	83.9	87.4	91	4.6	9.7
Sieve	2	-	45	88	-	-
MPTT	1	-	69.6	65.3	-	-
ESI	3	40 - 94.7	42.3 – 100	93.7 - 99	-	-

Data are presented as percentage. *: number of articles; #: overall accuracy. START: Simple Triage and Rapid Treatment; mSTART: Modified Simple Triage Algorithm and Rapid Treatment; SALT: Sort, Assess, Lifesaving interventions, Treatment/Transport; ASAV: Amberg-Schwandorf Algorithm for Primary Triage; MPTT: Modified Physiological Triage Tool; ESI: Emergency Severity Index.

## 5. Limitations

There were some limitations observed in conducting this research: 1) Studies have been done in different years. 2) They are not homogeneous, as they have examined populations with different age groups. 3) They have been conducted on people exposed to various incidents. 4) All triage systems are not studied in a single research. 5) Studies have not compared all the indicators of accuracy in a single research and 6) They have failed to conduct studies in real disaster situations. 7) Although 9 different triage systems were included in the study, few studies were found related to each system. For this reason, various aspects of triage systems in terms of accuracy have not been addressed adequately. 8) We just focused on articles published in English, other non-English articles were missed. 9) Articles included in the study were not homogenous in terms of study population, as some used real cases while others were done based on scenario-based incidents. 

## 6. Conclusion

According to the findings of the current systematic review, the performance of the existing Triage systems in terms of accuracy of prioritizing the injured people and other performance indexes is not desirable. Each country, usually based on its local context, chooses one of the triage systems or designs a new model. Iran does not have any local Hospital Triage for Disaster and Mass casualty incidents conditions and it is advisable to develop a new national model to address this issue in Iran. Therefore, to improve the performance and increase the precision of triage systems, the world nations are recommended to change or revise the indexes used in triage models and also identify other influential factors affecting the accuracy of triage systems. This not only makes the resources and facilities available for the injured needing lifesaving interventions, but also prevents wasting the limited medical resources and/or endangering human lives. 

## 7.2. Conflict of interest

The authors have declared that no competing interests exist.

## 7.3. Funding/Support

The author(s) received no financial support for the research, authorship and/or publication of this article.

## 7.4. Authors’ contribution

JB, MF, and HRK conceptualized the study questions and performed revisions. JB, AS, and HS performed the searches. JB, MF, AS, HS, and HRK conducted the statistical analyses. JB, and HS provided the draft of the manuscript. All authors have read and approved the final draft of the manuscript.

## 7.5. Ethical Considerations

This research was approved by Tehran University of Social Welfare and Rehabilitation Sciences and the code of ethics received was IR.USWR.REC.1398.07. All ethical principles were considered in this article. 
